# Janus kinase inhibitors for the treatment of rheumatoid arthritis demonstrate similar profiles of in vitro cytokine receptor inhibition

**DOI:** 10.1002/prp2.537

**Published:** 2019-11-15

**Authors:** Martin E. Dowty, Tsung H. Lin, Michael I. Jesson, Martin Hegen, David A. Martin, Vaibhav Katkade, Sujatha Menon, Jean‐Baptiste Telliez

**Affiliations:** ^1^ Pfizer Inc Cambridge MA USA; ^2^ Pfizer Inc Collegeville PA USA; ^3^ Pfizer Inc Groton CT USA

**Keywords:** cytokine receptors, immunopharmacology, inhibition, JAK‐STAT, rheumatoid arthritis

## Abstract

Janus kinase (JAK) inhibitors have emerged as an effective class of therapies for various inflammatory diseases such as rheumatoid arthritis (RA). JAK inhibitors function intracellularly by modulating the catalytic activity of JAKs and disrupting the receptor‐mediated signaling of multiple cytokines and growth factors, including those with pro‐inflammatory activity. Understanding the inhibition profiles of different JAK inhibitors, based on the associated cytokine receptors and downstream inflammatory pathways affected, is important to identify the potential mechanisms for observed differences in efficacy and safety. This study applied an integrated modeling approach, using in vitro whole blood cytokine inhibition potencies and plasma pharmacokinetics, to determine JAK‐dependent cytokine receptor inhibition profiles, in the context of doses estimated to provide a similar clinical response in RA clinical trials. The calculated profiles of cytokine receptor inhibition for the JAK inhibitors tofacitinib, baricitinib, upadacitinib, and filgotinib and its metabolite, were generally similar when clinically efficacious doses for RA were considered. Only minor numerical differences in percentage cytokine receptor inhibition were observed, suggesting limited differentiation of these inhibitors based on JAK pharmacology, with each showing a differential selectivity for JAK1 heterodimer inhibition. Nevertheless, only robust clinical testing involving head‐to‐head studies will ultimately determine whether there are clinically meaningful differences between these JAK inhibitors. Furthermore, ongoing and future research into inhibitors with alternative JAK selectivity remains of clinical importance. Thus, all JAK inhibitors should be characterized via thorough preclinical, metabolic and pharmacological evaluation, adequate long‐term clinical data, and when available, real‐world experience.

**Abbreviations**
ACR: American College of Rheumatology
ATP: adenosine triphosphate
BID: twice daily
C_av_: average plasma concentration
C_av,u_: average plasma concentration, unbound
CD: cluster of differentiation
CI: confidence interval
CRP: C‐reactive protein
EDTA: ethylenediaminetetraacetic acid
EMA: European Medicines Agency
EPO: erythropoietin
FDA: United States Food and Drug Administration
f_u_: fraction unbound
G‐CSF: granulocyte colony‐stimulating factor
GM‐CSF: granulocyte‐macrophage colony‐stimulating factor
HZ: herpes zoster
IC_50_: half‐maximal inhibitory concentration
IC_50,u_: half‐maximal inhibitory concentration, unbound
IC_xx_: proportion of JAK inhibitory concentration
IFN: interferon
IL: interleukin
IR: incidence rate
JAK: Janus kinase
K_M_: Michaelis‐Menten constant
LC‐MS/MS: liquid chromatography‐tandem mass spectrometry
NA: not applicable
NK: natural killer
PBS: phosphate‐buffered saline
pSTAT: phosphorylated signal transducer and activator of transcription protein
QD: once daily
RA: rheumatoid arthritis
RFU: relative fluorescence unit
SD: standard deviation
TPO: thrombopoietin
TYK: tyrosine kinase

## 1 INTRODUCTION

Inflammatory diseases, such as rheumatoid arthritis (RA), are chronic immune‐mediated conditions, which collectively have an estimated prevalence of 5%‐7% in Western society,^1^ can be highly disabling^2^ and painful, and cause reduced quality of life.^3^

Inflammation is a complex immune response involving diverse cell types and feedback loops promoting the production of pro‐inflammatory mediators.^1^, ^4^ Notably, cytokines are a group of structurally unrelated protein messengers that, upon binding to and activating their specific receptors on immune cells, transmit signals that regulate the expression of numerous inflammatory genes. Under dysregulated conditions, cytokines play a key role in the pathologic inflammatory response characteristic of immune‐mediated diseases.^1^, ^5^, ^6^

Janus kinases (JAKs) are enzymes that are essential in the signaling pathways of type I and type II cell‐surface cytokine receptors which lack intrinsic kinase catalytic activity.^5^, ^6^ There are four different JAK isoforms in humans (JAK1, JAK2, JAK3, and TYK2) which function in pairs to transmit intracellular signals from cytokine‐activated receptors.^5^ JAK1 pairs with JAK3 to mediate the signaling pathways of common gamma chain (γc) cytokines. JAK1 also pairs with JAK2 and/or TYK2 for signaling through receptors of the IL‐6, IL‐10, and interferon cytokine families. Additionally, JAK2 pairs with TYK2 for signaling through IL‐12 and IL‐23 cytokine receptors, and with itself for signaling from receptors for hormone‐like cytokines such as erythropoietin (EPO).^5^

Indeed, there are over 50 cytokines that signal through JAK‐mediated type I and II receptors,^6^ many implicated in inflammatory disease pathophysiology.^1^, ^4^ Notably, IL‐6 induces acute‐phase proteins such as C‐reactive protein (CRP) and may be involved in the autoimmune process through B‐cell modulation and T‐helper‐17‐cell differentiation.^7^ Common γc cytokines play a key role in adaptive immune functions, for example, in T‐cell and natural killer (NK)‐cell differentiation.^5^ JAKs are therefore an attractive therapeutic target for RA and other inflammatory diseases.^6^

Biologic drugs for treating inflammatory diseases target extracellular elements of the inflammation pathway such as cytokines or their receptors.^1^, ^4^, ^5^ In contrast, targeted synthetic JAK inhibitors reduce inflammation by directly binding to and modulating the intracellular catalytic activity of JAKs and disrupting the receptor‐mediated signaling of multiple cytokines, including those of pro‐inflammatory pathways.^4^

Current JAK inhibitor drugs were designed to be selective for certain JAK isoforms.^8^ Tofacitinib is an oral, small molecule JAK inhibitor for the treatment of RA, psoriatic arthritis, and ulcerative colitis. Tofacitinib is reported to have functional selectivity for heterodimer pairs involving JAK1 and/or JAK3.^4^, ^5^ Other JAK inhibitors with ongoing or completed late development RA clinical trials include baricitinib, upadacitinib, and filgotinib. Baricitinib is approved for the treatment of RA, with reported preferential selectivity for JAK1 and JAK2.^9^ Upadacitinib and filgotinib are under investigation for the treatment of inflammatory diseases including RA; both drugs, as well as filgotinib's active metabolite, are reported to selectively inhibit JAK1.^10^, ^11^, ^12^

Understanding the different inhibition profiles of JAK inhibitors, based on the associated cytokine receptors and downstream inflammatory pathways inhibited, is important to better characterize the impact of JAK inhibition and the potential rationale for differences in clinical efficacy and safety profiles. Although JAK selectivity can be evaluated using enzymatic assays, the selectivity observed using biochemical assays may not necessarily be maintained when evaluated under physiologic cellular conditions.^13^ Suggested reasons for this discrepancy include differences within the complex intracellular milieu, particularly when assessing activity in primary cells (ie, human whole blood), such as the difference in the adenosine triphosphate (ATP) Michaelis‐Menten constant (K_M_) of each kinase.^13^

The objective of this study was to characterize cytokine receptor inhibition profiles of JAK inhibitors for the treatment of RA, in the context of drug doses that provided a similar response in patients in RA clinical trial settings. To achieve this, we applied an integrated modeling approach, using knowledge of both intracellular JAK‐dependent cytokine signaling inhibition potencies and in vivo pharmacokinetics.

## 2 MATERIALS AND METHODS

### 2.1 Drug compounds

Tofacitinib and the active metabolite of filgotinib were synthesized by Pfizer Discovery Research. Baricitinib (Catalog No. G‐5743), upadacitinib (Catalog No. M15685), and filgotinib (Catalog No. I‐9794) were purchased from Advanced ChemBlocks Inc. The compounds were prepared as 30 mmol/L stocks in 100% dimethyl sulfoxide (DMSO). An 11‐point dilution series was created in DMSO with a maximum concentration of 10 mmol/L or 30 mmol/L. Further dilution was performed by adding 4 µL of the above compound solutions into 96 µL of phosphate‐buffered saline (PBS) with a maximum concentration of 400 µmol/L or 1200 µmol/L.

### 2.2 Cytokines

EPO (Catalog No. 287 TC), G‐CSF (Catalog No. 214‐GS), GM‐CSF (Catalog No. 215‐GM), IFNα (Catalog No. 11200‐2), IFNγ (Catalog No. 285‐IF), IL‐2 (Catalog No. 202‐IL), IL‐3 (Catalog No. 203‐IL), IL‐4 (Catalog No. 204‐IL), IL‐6 (Catalog No. 206‐IL), IL‐7 (Catalog No. 207‐IL), IL‐10 (Catalog No. 217‐IL), IL‐12 (Catalog No. 219‐IL), IL‐13 (Catalog No. 213‐IL), IL‐15 (Catalog No. 247‐IL), IL‐23 (Catalog No. 1290‐IL), IL‐27 (Catalog No. 2526‐IL), and TPO (Catalog No. 288‐TP) were obtained from R&D Systems. IL‐21 (Catalog No. AF‐200‐21) was purchased from PeproTech.

### 2.3 Antibodies

Antibodies specific to phosphorylated signal transducer and activator of transcription proteins (pSTATs) and cluster of differentiation (CD) molecules were supplied by BD Biosciences (San Jose, CA, USA): anti‐pSTAT1‐AF647 (Catalog No. 612597); anti‐pSTAT1‐AF488 (Catalog No. 612596); anti‐pSTAT3‐AF647 (Catalog No. 557815); anti‐pSTAT4‐AF647 (Catalog No. 558137); anti‐pSTAT5‐AF488 (Catalog No. 612598); anti‐pSTAT5‐AF647 (Catalog No. 612599); anti‐CD3‐BV421 (Catalog No. 562426); anti‐CD3‐BV650 (Catalog No. 563852); anti‐CD14‐Pacific Blue (Catalog. No. 558121); anti‐CD14‐AF488 (Catalog No. 557700); anti‐CD19‐BV421 (Catalog No. 562440).

### 2.4 Cells

Cryopreserved human bone marrow CD34^+^ cells were purchased from STEMCELL Technologies (Catalog No. 70002.3; Vancouver, Canada). Frozen bone marrow CD34^+^ cells were thawed, washed once with StemSpan™ SFEM II medium (Catalog No. 09605; STEMCELL Technologies), suspended in StemSpan™ SFEM II medium containing StemSpan™ Erythroid Expansion Supplement (Catalog No. 02692; STEMCELL Technologies), and cultured for 7 days. CD34^+^ cells were then harvested, washed once with Dulbecco's (D)‐PBS, and suspended at 0.5 x 10^6^ cells/mL in human whole blood to be used in the EPO stimulation assay.

Human whole blood was collected from 13 healthy donors (seven males and six females) via venipuncture into Vacutainer collection tubes containing sodium heparin, in accordance with Pfizer protocols (Protocol No. GOHW RDP‐01) approved by the Shulman Institutional Review Board. Blood was warmed to 37°C prior to use.

### 2.5 Other materials

Phosflow Lyse/Fix Buffer 5X (Catalog No. 558049) was purchased from BD Biosciences. Fetal bovine serum (Catalog No. A3160601) was purchased from Thermo Fisher Scientific and sodium azide (Catalog No. S8032) was obtained from Sigma Aldrich. D‐PBS (without Ca^2+^ or Mg^2+^) was obtained from Invitrogen (Catalog No. 14190). Glutathione S‐transferase (GST)‐tagged recombinant human kinase domains of JAK1, JAK2, and JAK3 were purchased from Thermo Fisher Scientific. His‐tagged recombinant human TYK2 kinase domain was expressed in SF21/baculovirus and purified using a two‐step affinity (Ni‐nitrilotriacetic acid) and size‐exclusion (SEC S200) purification method.

### 2.6 Enzymatic potency of JAK inhibitors

The potency of tofacitinib, upadacitinib, baricitinib, and filgotinib and its metabolite against the four JAK isoforms, JAK1, JAK2, JAK3, and TYK2, was measured in terms of half‐maximal inhibitory concentration (IC_50_). Human JAK activity was determined using a microfluidic assay to monitor phosphorylation of a synthetic peptide by the recombinant human kinase domain of each of the JAK isoforms.

Test compounds were solubilized in DMSO to a stock concentration of 30 mmol/L. Compounds were diluted in DMSO to create an 11‐point half‐log dilution series with a maximum concentration of 600 μmol/L. The test compound plate also contained positive control wells containing a proprietary potent inhibitor to define 100% inhibition and negative control wells containing DMSO to define no inhibition. The test compounds were diluted 1:60 in the assay, resulting in a final assay compound concentration range of 10 μmol/L to 100 pmol/L, with a final assay concentration of 1.7% DMSO. Test compounds and controls solubilized in 100% DMSO were added (250 nL) to a 384‐well polypropylene plate (Corning) using a non‐contact acoustic dispenser.

Kinase assays were carried out at room temperature in 15 μL reaction buffer containing 20 mmol/L HEPES (pH 7.4), 10 mmol/L magnesium chloride, 0.01% bovine serum albumin, 0.0005% Tween 20, and 1 mmol/L dithiothreitol. Reaction mixtures contained 1 μmol/L of a fluorescently labeled synthetic peptide (5FAM‐KKSRGDYMTMQID for JAK1 and TYK2, and FITC‐KGGEEEEYFELVKK for JAK2 and JAK3) at a concentration less than the apparent K_M_. Reaction mixtures contained 1 mmol/L ATP.

Test compound was added to the buffer containing ATP and substrate, and immediately after this step, the enzyme was added to begin the reaction. The assays were stopped with 15 μL of a buffer containing 180 mmol/L HEPES (pH = 7.4), 20 mmol/L ethylenediaminetetraacetic acid (EDTA), and 0.2% coating reagent, resulting in a final concentration of 10 mmol/L EDTA, 0.1% coating reagent, and 100 mmol/L HEPES (pH = 7.4). The assay plates were placed on a Caliper Life Science Lab Chip 3000 (LC3000) instrument or Caliper Life Science EZ Reader instrument and each well was sampled using appropriate separation conditions to determine the level of phosphorylation.

### 2.7 In vitro analyses

#### 2.7.1 Whole blood potency

Inhibition curves and IC_50_ values were determined for cytokine signaling of tofacitinib, baricitinib, upadacitinib, and filgotinib and its metabolite. For each cytokine assay, all five compounds were tested side‐by‐side in quadruplicate (ie, using blood from four donors).

A total of 90 µL/well of human whole blood was added to 96‐well polypropylene plates (Catalog No. 10755‐246; VWR), followed by 5 µL test compound solutions prepared per above to give a maximum concentration of 20 µmol/L or 60 µmol/L. The plates were mixed and incubated for 60 minutes at 37ºC. Then, 5 µL of PBS or cytokine was added to each well (5 µL/well; final, 10 U/mL EPO, 100 ng/mL G‐CSF, 10 ng/mL GM‐CSF, 5000 U/mL IFNα, 100 ng/mL IFNγ, 50 ng/mL IL‐2, 50 ng/mL IL‐3, 5 ng/mL IL‐4, 50 ng/mL IL‐6, 30 ng/mL IL‐7, 30 ng/mL IL‐10, 30 ng/mL IL‐12, 30 ng/mL IL‐13, 30 ng/mL IL‐15, 50 ng/mL IL‐21, 25 ng/mL IL‐23, 1000 ng/mL IL‐27) and incubated for 15 minutes. Anti‐cell surface antibodies were added 15 minutes prior to cytokine stimulation; anti‐CD14‐Pacific Blue (0.5 µL/well) to GM‐CSF, IFNγ, and TPO‐treated samples; anti‐CD3‐BV650 (0.5 µL/well) plus anti‐CD14‐Pacific Blue (0.5 µL/well) to IL‐6‐treated samples; and anti‐CD19‐BV421 (0.5 µL/well) plus anti‐CD14‐AF488 (0.5 µL/well) to IL‐13‐treated samples.

The reaction was quenched by adding Lyse/Fix Buffer to all wells at 700 µL/well and incubating for 20 minutes at 37°C; after washing with staining buffer (D‐PBS containing 0.5% fetal bovine serum and 0.1% sodium azide), 350 µL ice‐cold 90% methanol (Catalog No. 4823‐32; RICCA Chemical) was added to each well and incubated at 4ºC for 30 minutes. One more wash was done with staining buffer and all samples were finally suspended in 150 µL/well of the desired anti‐pSTAT antibodies at 1:150 dilution in staining buffer; anti‐pSTAT1‐AF647 in IFNγ‐treated samples; anti‐pSTAT1‐AF488 and anti‐pSTAT3‐AF647 in IFNα‐, IL‐6‐, and IL‐27‐treated samples; anti‐pSTAT3‐AF647 in G‐CSF‐, IL‐10‐, IL‐21‐, and IL‐23‐treated samples; anti‐pSTAT4‐AF647 in IL‐12‐treated samples; anti‐pSTAT5‐AF647 in EPO‐, GM‐CSF‐, IL‐2‐, IL‐3‐, IL‐7‐, IL‐15‐, and TPO‐treated samples; anti‐pSTAT5‐AF488 and anti‐pSTAT6‐AF647 in IL‐4‐treated samples; and anti‐pSTAT6‐AF647 in IL‐13‐treated samples.

After overnight incubation at 4°C, all the samples were transferred into 96‐well polypropylene U‐bottom plates (Catalog No. 072‐00‐745; Thermo Fisher Scientific) and flow cytometric analysis was performed on an LSR Fortessa equipped with a High Throughput Sampler plate loader (BD Biosciences). The lymphocyte population was gated for pSTAT histogram analysis for IFNα‐, IL‐2‐, IL‐3‐, IL‐4‐, IL‐7‐, IL‐10‐, IL‐12‐, IL‐15‐, IL‐21‐, IL‐23‐, and IL‐27‐treated samples; granulocyte population for G‐CSF‐treated samples; CD14^+^ cells for GM‐CSF‐, IFNγ‐, and TPO‐treated samples; CD3^+^ cells and CD14^+^ cells for IL‐6‐treated samples; CD14^+^ cells and CD19^+^ cells for IL‐13‐treated samples; all events (entire populations) for EPO‐treated cells. Background fluorescence was defined using unstimulated cells and a gate was placed at the foot of the peak to include ~0.5% gated population.

Histogram statistical analysis was performed using FACSDiva version 8.0.1 (BD Biosciences). Relative fluorescence unit (RFU), which measures the level of pSTAT, was calculated by multiplying the percent positive population and its mean fluorescence. Data from 11 compound concentrations (singlicate at each concentration) were normalized as a percentage of control, as shown in Equation 1:

(1) $$\%\;\text{of}\;\text{control}=100\times \frac{\left(A-B\right)}{\left(C-B\right)}$$

*A*, RFU from wells containing compound and cytokine; *B*, RFU from wells without cytokine (minimum fluorescence); *C*, RFU from wells containing only cytokine (maximum fluorescence).

Inhibition curves and IC_50_ values were determined using Prism software (Version 7, GraphPad).

#### 2.7.2 Plasma protein binding

An equilibrium dialysis method was used to determine the plasma fraction unbound (f_u_) values, as described previously.^14^ Briefly, dialysis membranes (MWCO 12‐14K) and 96‐well dialysis devices were assembled following the manufacturer's instructions (HTDialysis, LLC, Gales Ferry, CT, USA). Human plasma samples (pooled mixed gender; BioIVT, www.bioivt.com) containing 1 μmol/L test compounds with 1% DMSO were dialyzed against PBS for 6 hours in a humidified incubator (75% relative humidity; 5% CO_2_/95% air) at 37°C with shaking at 450 RPM. Quadruplicates of binding were measured for each compound. Samples were matrix‐matched and quenched with cold acetonitrile containing internal standard(s). The solutions were centrifuged and the supernatant was analyzed using liquid chromatography‐tandem mass spectrometry (LC‐MS/MS).

#### 2.7.3 Blood‐to‐plasma ratio

Human blood‐to‐plasma ratio was measured by Unilabs York Bioanalytical Solutions. Test compounds were incubated in quadruplicate with fresh human blood (mixed gender, at least 1 sample per gender, Clinical Trials Laboratory Services Ltd, London, UK) at 1 μmol/L in a humidified incubator (95% relative humidity; 5% CO_2_/95% air) for 1 and 3 hours at 37°C with shaking at 450 RPM. Following incubation, plasma samples were obtained by centrifuging blood samples at 3000*g* for 7 minutes. Both plasma and blood samples were matrix‐matched with each other and quenched with acetonitrile containing internal standard. The solutions were centrifuged, and the supernatant was analyzed by LC‐MS/MS. Peak area ratios were used to calculate blood‐to‐plasma ratio.

### 2.8 Data analyses

An integrated modeling approach was applied to determine cytokine receptor inhibition profiles. Whole blood IC_50_ values were converted to unbound values (IC_50,u_) using measured blood‐to‐plasma ratios and measured f_u_ values, as shown in Equation 2:

(2) $${\text{IC}}_{50,u}=\frac{{\text{IC}}_{50}}{\text{blood}:\text{plasma ratio}}\times f_{u}$$

Human daily average plasma concentrations (C_av_) were used as reported or predicted in the literature for tofacitinib, baricitinib, upadacitinib, and filgotinib and its metabolite, for doses that we determined to be clinically meaningful (ie, tofacitinib 5 mg twice daily [BID], baricitinib 4 mg once daily [QD], upadacitinib 15 mg QD, and filgotinib 200 mg QD). Doses were selected on the rationale that they provided a generally comparable proportion of patients meeting American College of Rheumatology (ACR) response criteria in clinical trial settings.^15^, ^16^, ^17^, ^18^ C_av_ as a measure of average daily drug plasma concentration was used as it has been shown to be a predictive exposure metric of the efficacy of tofacitinib and filgotinib, rather than C_max_ or C_min_.^19^, ^20^, ^21^ C_av_ values were converted to unbound values (C_av,u_) using f_u_ values, as shown in Equation 3:

(3) $$C_{\text{av,u}}=C_{\text{av}}\times f_{u}$$

Proportions (%) of JAK receptor inhibitory concentrations (IC_xx_) at clinically meaningful doses were then calculated, as shown in Equation 4:

(4) $${\text{IC}}_{\text{xx}}=100\times \frac{C_{\text{av},u}}{{\text{IC}}_{50,u}+C_{\text{av},u}}$$

## 3 RESULTS

### 3.1 Enzymatic potency of JAK inhibitors

Table 1 presents IC_50_ values for the inhibition of the JAK isoforms (JAK1, JAK2, JAK3, and TYK2) by tofacitinib, baricitinib, upadacitinib, and filgotinib, measured using an enzymatic assay. Measurements were performed once for the filgotinib metabolite and showed weak inhibition (therefore data not shown).

**Table 1:** Mean IC_50_ values in enzymatic assay for tofacitinib, baricitinib, upadacitinib, and filgotinib inhibition of JAK1, JAK2, JAK3, and TYK2

	IC_50_ (nmol/L) [n]^a^, ^b^			
	JAK1	JAK2	JAK3	TYK2
Tofacitinib	15 [7]	71 [7]	45 [8]	472 [10]
Baricitinib	0.78 [3]	2 [3]	253 [3]	14 [3]
Upadacitinib	0.76 [3]	19 [3]	224 [3]	118 [3]
Filgotinib	45 [3]	357 [3]	9097 [3]	397 [3]

Abbreviations: ATP, adenosine triphosphate; IC_50_, half‐maximal inhibitory concentration; JAK, Janus kinase; TYK, tyrosine kinase.

a IC_50_ values represent the geometric mean of independent experiments; [n] denotes the number of experiments.

b All reactions were carried out in the presence of 1 mmol/L ATP.

### 3.2 In vitro potency of JAK inhibitors

#### 3.2.1 Whole blood potency

Table 2 presents mean IC_50_ values for inhibition of cytokine receptors by tofacitinib, baricitinib, upadacitinib, and filgotinib and its metabolite, measured in human whole blood side‐by‐side. Measurements were performed for each compound at 11 different concentrations, and using blood from four donors per cytokine. Additionally, Figure S1 presents IC_50_ curves (one of the four obtained) for representative cytokine receptors from different receptor classes (IFNγ, IFNα, IL‐6, IL‐15, IL‐12, and EPO) for illustrative purposes.

**Table 2:** Mean IC_50_ and IC_50,u_ values in human whole blood from four donors for tofacitinib, baricitinib, upadacitinib, and filgotinib

JAK signaling pair	Gated cell population	STAT phosphorylation	Cytokine receptor	Tofacitinib^a^		Baricitinib^b^		Upadacitinib^c^		Filgotinib^d^		Filgotinib metabolite^e^	
				Mean (SD) human whole blood, nmol/L									
				IC_50_	IC_50,u_	IC_50_	IC_50,u_	IC_50_	IC_50,u_	IC_50_	IC_50,u_	IC_50_	IC_50,u_
JAK1/JAK2	CD14^+^ cells	pSTAT1	IFNγ	225 (18)	114	109 (12)	49	109 (11)	53	6953 (568)	2792	>60 000 (NA)	
JAK1/JAK2	Granulocytes	pSTAT3	G‐CSF	247 (12)	125	146 (17)	65	78 (11)	38	6108 (900)	2453	>52 300 (NA)	
JAK1/TYK2	Lymphocytes	pSTAT1	IFNα	58 (7.8)	29	46 (5.6)	21	21 (1.5)	10	1323 (155)	531	11 525 (826)	4441
JAK1/TYK2	Lymphocytes	pSTAT3	IFNα	48 (8.2)	24	38 (5.9)	17	19 (2.2)	9.2	1057 (143)	425	9880 (1260)	3807
JAK1/TYK2	Lymphocytes	pSTAT3	IL‐10	126 (62)	64	90 (43)	40	90 (31)	43	2550 (1083)	1024	>42 100 (NA)	
JAK1/JAK2, JAK1/TYK2	CD3^+^ cells	pSTAT1	IL‐6	30 (4.4)	15	21 (1.8)	9.2	12 (0.47)	5.9	671 (131)	270	7230 (1169)	2786
JAK1/JAK2, JAK1/TYK2	CD3^+^ cells	pSTAT3	IL‐6	340 (38)	173	229 (27)	102	152 (26)	73	4938 (425)	1983	>54 700 (NA)	
JAK1/JAK2, JAK1/TYK2	CD14^+^ cells	pSTAT1	IL‐6	48 (9.0)	24	33 (5.3)	15	26 (3.9)	13	810 (207)	325	7583 (1158)	2922
JAK1/JAK2, JAK1/TYK2	CD14^+^ cells	pSTAT6	IL‐13	275 (30)	140	196 (24)	88	126 (16)	61	5143 (709)	2065	>60 000 (NA)	
JAK1/JAK2, JAK1/TYK2	CD19^+^ cells	pSTAT6	IL‐13	119 (30)	61	73 (17)	33	72 (13)	35	2125 (359)	853	40 250 (11 656)	15 509
JAK1/JAK2, JAK1/TYK2	Lymphocytes	pSTAT1	IL‐27	73 (9.5)	37	34 (4.2)	15	52 (4.2)	25	2113 (187)	848	21 525 (3351)	8294
JAK1/JAK2, JAK1/TYK2	Lymphocytes	pSTAT3	IL‐27	56 (9.3)	29	30 (5.4)	13	44 (6.4)	21	1165 (121)	468	9605 (1113)	3701
JAK1/JAK3	Lymphocytes	pSTAT5	IL‐2	54 (6.0)	27	90 (18)	40	50 (8.3)	24	2660 (611)	1068	28 650 (5991)	11 039
JAK1/JAK3	Lymphocytes	pSTAT5	IL‐4	11 (2.7)	5.4	14 (3.2)	6.1	8.2 (3.7)	4.0	363 (140)	146	4968 (1891)	1914
JAK1/JAK3	Lymphocytes	pSTAT6	IL‐4	84 (23)	42	131 (36)	59	77 (20)	37	3623 (897)	1455	>54 300 (NA)	
JAK1/JAK3	Lymphocytes	pSTAT5	IL‐7	72 (23)	36	126 (46)	56	101 (31)	49	3625 (988)	1456	41600 (9523)	16 029
JAK1/JAK3	Lymphocytes	pSTAT5	IL‐15	67 (1.9)	34	110 (15)	49	64 (6.2)	31	3378 (980)	1357	31700 (8358)	12215
JAK1/JAK3	Lymphocytes	pSTAT3	IL‐21	79 (14)	40	142 (16)	63	51 (5.0)	25	3985 (278)	1601	>48700 (NA)	
JAK2/TYK2	Lymphocytes	pSTAT4	IL‐12	265 (93)	134	91 (26)	41	145 (53)	70	8028 (2149)	3224	>5900 (NA)	
JAK2/TYK2	Lymphocytes	pSTAT3	IL‐23	408 (135)	207	115 (39)	51	242 (79)	117	13193 (3470)	5299	>58100 (NA)	
JAK2/JAK2	Erythroid progenitor cells	pSTAT5	EPO	290 (88)	148	105 (33)	47	174 (66)	84	9895 (3481)	3974	53 550 (3748)	2034
JAK2/JAK2	CD14^+^ cells	pSTAT5	TPO	608 (189)	309	243 (42)	109	297 (66)	143	>17 600 (NA)		>60 000 (NA)	
JAK2/JAK2	Lymphocytes	pSTAT5	IL‐3	825 (54)	419	309 (23)	138	326 (33)	158	>20 000 (NA)		>6000 (NA)	
JAK2/JAK2	CD14^+^ cells	pSTAT5	GM‐CSF	1758 (237)	893	637 (110)	285	782 (138)	377	>20 000 (NA)		>60 000 (NA)	

Note

Missing data for filgotinib are due to the IC_50_ values not being measurable.

Abbreviations: CD, cluster of differentiation; EPO, erythropoietin; f_u_, fraction unbound; G‐CSF, granulocyte colony‐stimulating factor; GM‐CSF, granulocyte‐macrophage colony‐stimulating factor; IC_50_, half‐maximal inhibitory concentration; IC_50,u_, half‐maximal inhibitory concentration, unbound; IFN, interferon; IL, interleukin; JAK, Janus kinase; NA, not applicable; pSTAT, phosphorylated signal transducer and activator of transcription protein; SD, standard deviation; TPO, thrombopoietin; TYK, tyrosine kinase.

a Blood‐to‐plasma ratio, 1.20; f_u_, 0.61.

b Blood‐to‐plasma ratio, 1.32; f_u_, 0.59.

c Blood‐to‐plasma ratio, 1.16; f_u_, 0.56.

d Blood‐to‐plasma ratio, 1.22; f_u_, 0.49.

e Blood‐to‐plasma ratio, 1.09; f_u_, 0.42.

#### 3.2.2 Plasma protein binding

From quadruplicate measurements, mean f_u_ values were determined to be 0.61 for tofacitinib, 0.59 for baricitinib, 0.56 for upadacitinib, 0.49 for filgotinib, and 0.42 for filgotinib metabolite.

#### 3.2.3 Blood‐to‐plasma ratios

From quadruplicate measurements, mean blood‐to‐plasma ratios were determined to be 1.20 for tofacitinib, 1.32 for baricitinib, 1.16 for upadacitinib, 1.22 for filgotinib, and 1.09 for filgotinib metabolite.

#### 3.2.4 Unbound half‐maximal inhibitory concentrations

Per Equation 2, the measured IC_50_ values, f_u_ values, and blood‐to‐plasma ratios were used to calculate IC_50,u_ values, which are presented alongside the IC_50_ values in Table 2.

### 3.3 Cytokine inhibition

Table 3 presents C_av_ values obtained from the literature for doses that were determined to be clinically meaningful (based on generally comparable ACR response rates), and calculated C_av,u_ values (Equation 3) for tofacitinib, baricitinib, upadacitinib, and filgotinib and its metabolite.

**Table 3:** Actual and unbound C_av_ values for clinically meaningful doses of tofacitinib, baricitinib, upadacitinib, and filgotinib and its metabolite

	Dose	C_av_ (nmol/L)	C_av,u_ (nmol/L)	f_u_
Tofacitinib^19^	5 mg BID^a^	68	41.0	0.61
Baricitinib^9^	4 mg QD^b^	27	16.0	0.59
Upadacitinib^47^, ^48^	15 mg QD^c^	49	27.4	0.56
Filgotinib^11^	200 mg QD^c^	474	232.0	0.49
Filgotinib metabolite^11^	200 mg QD^c^	7438	3124.0	0.42

Abbreviations: BID, twice daily; C_av_, average plasma concentration; C_av,u_, average plasma concentration, unbound; EMA, European Medicines Agency; FDA, United States Food and Drug Administration; f_u_, fraction unbound; QD, once daily.

a This dose is approved by both the EMA and the FDA.

b This dose is approved by the EMA but not approved by the FDA (dosage of 2 mg QD is approved by the FDA).

c These JAK inhibitors are under investigation.

**Figure 1:**
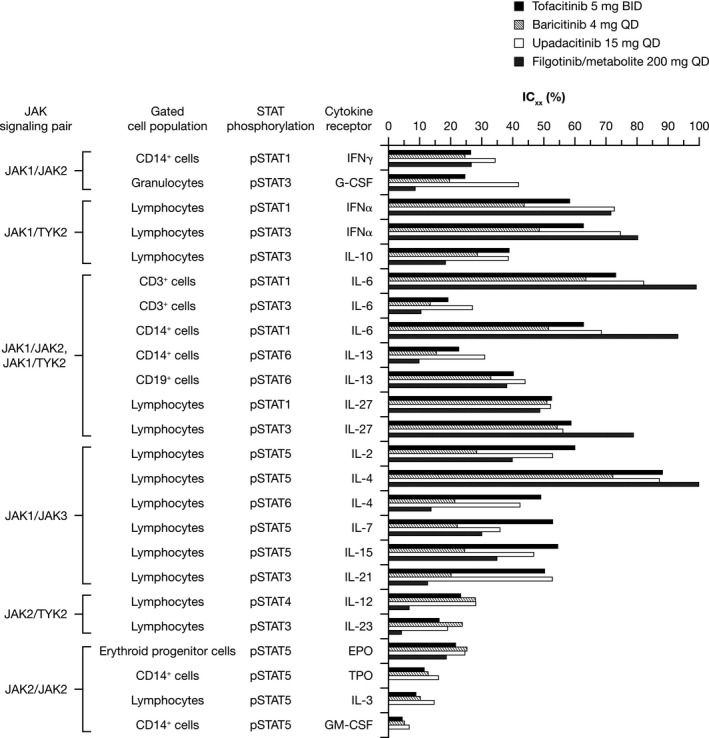
Cytokine receptor inhibitory concentrations for modeled exposures of tofacitinib 5 mg BID, baricitinib 4 mg QD, upadacitinib 15 mg QD, and filgotinib/metabolite 200 mg QD. BID, twice daily; CD, cluster of differentiation; EPO, erythropoietin; G‐CSF, granulocyte colony‐stimulating factor; GM‐CSF, granulocyte‐macrophage colony‐stimulating factor; IC_xx_, proportion of JAK inhibitory concentration; IFN, interferon; IL, interleukin; JAK, Janus kinase; pSTAT, phosphorylated signal transducer and activator of transcription protein; QD, once daily; TPO, thrombopoietin; TYK, tyrosine kinase

Calculated IC_xx_ data (Equation 4) are summarized in Figure 1. Cytokine receptor inhibition profiles across a broad range of pathways were generally similar among the JAK inhibitors studied, and generally consistent across each JAK pair. There appeared to be some small numerical differences among the JAK inhibitors analyzed. Relative inhibition of most JAK1/3‐mediated cytokine receptors (γc cytokine receptors; IL‐2, IL‐4, IL‐7, and IL‐15) appeared to be numerically greater with tofacitinib vs other JAK inhibitors; inhibition of JAK2/TYK2‐mediated cytokine receptors (IL‐12, IL‐23, and EPO) appeared to be numerically greater with baricitinib vs other JAK inhibitors; and inhibition of JAK1/JAK2‐mediated cytokine receptors (IFNγ and G‐CSF) and JAK2‐mediated cytokine receptors (TPO, IL‐3, and GM‐CSF) appeared to be numerically greater with upadacitinib vs other JAK inhibitors.

## 4 DISCUSSION

The relationship between the clinical profiles of JAK inhibitors and their individual selectivity for JAK isoforms is not clear. To explore this, we evaluated the activity of JAK inhibitors approved or under investigation for RA treatment. Using an integrated modeling approach, we determined cytokine receptor inhibition profiles of clinically equivalent doses (based on proportion of patients meeting ACR response criteria) of tofacitinib (5 mg BID), baricitinib (4 mg QD), upadacitinib (15 mg QD), and filgotinib and its metabolite (200 mg QD).

The IC_50_ values determined in this study were consistent with the literature, with each JAK inhibitor demonstrating greater selectivity for JAK1 vs other isoforms.^4^, ^5^, ^9^, ^10^, ^11^ JAK1 is presumed to be a key target for RA and other inflammatory diseases since it associates with receptors for γc cytokines, interferons, type II cytokine receptors (eg, IL‐6), and other interleukins.^5^, ^22^ Also per the literature, tofacitinib showed comparative selectivity for JAK3, and baricitinib showed comparative selectivity for JAK2.^4^, ^5^, ^9^

Cytokine receptor inhibitory concentration (IC_xx_) profiles were generally similar among the JAK inhibitors, with small numerical differences in percentage cytokine receptor inhibition, suggesting limited differentiation of these JAK inhibitors based on in vitro pharmacology. Small differences observed included the IC_xx_ values for IL‐6 and IL‐15 receptor inhibition. However, these differences do not appear to translate into significant differences in key clinical biomarkers. Although IL‐6 is involved in stimulating CRP production from hepatocytes,^7^ reduction of CRP levels from baseline to week 12 does not appear to differ by any clinically meaningful extent for these JAK inhibitors when used to treat RA: tofacitinib 5 mg BID (−10.1 mg/L^23^); baricitinib 4 mg QD (approximately −10 mg/L^24^); upadacitinib 6 mg BID (−8.8 mg/L^25^); and filgotinib 200 mg QD (−14.9 mg/L^26^). Likewise, although IL‐15 is critical for NK cell maintenance,^27^ reductions from baseline in NK cell counts do not appear to differ to a meaningful extent for these JAK inhibitors in RA: tofacitinib (−32.5 cells/mm^3^ at month 1.5, −63.5 cells/mm^3^ at month 6, +6.5 cells/mm^3^ at month 22, cross‐sectional analysis in different groups of patients^4^); baricitinib 4 mg QD (−57.0 cells/mm^3^ at week 12; −53.4 cells/mm^3^ at week 24^28^); upadacitinib 6 mg BID (approximately −50 cells/mm^3^ at week 12^29^); sufficient long‐term data are not available for filgotinib.

Tofacitinib generally demonstrated a greater relative inhibition of γc cytokine receptors vs the other JAK inhibitors. This is consistent with the selectivity of tofacitinib for JAK1 and JAK3,^4^, ^5^ compared with smaller JAK3 effects for the other JAK inhibitors.^9^, ^10^, ^11^ Given the association between γc cytokines and adaptive immune functions, including effects on subsets of pathologic T cells,^5^ one might expect to observe related clinical differences between these JAK inhibitors. It remains to be seen if the relative difference in inhibition of γc cytokine receptors vs other cytokine receptors (eg, IFN and IL‐6) with tofacitinib, compared with JAK inhibitors that spare JAK3, translates into a meaningful difference in infection risk. Meta‐analysis data suggest that the risk of serious infections is comparable for tofacitinib and baricitinib in the treatment of RA.^30^ Incidence rates (IRs; patients with events per 100 patient‐years) (95% confidence intervals [CI]) were: 2.7 (2.5, 3.0) for all tofacitinib doses pooled in phase 1, phase 2, phase 3, and long‐term extension (LTE) studies; and 4.8 (2.3, 9.7) for baricitinib 2 mg QD, and 3.7 (2.3, 5.8) for baricitinib 4 mg QD, in randomized controlled trials.^30^ Interestingly, herpes zoster (HZ), caused by varicella zoster virus reactivation,^31^ has been observed with each JAK inhibitor studied herein. In pooled analyses of RA clinical studies, HZ IRs (events per 100 patient‐years) were: 4.4 and 4.2 with tofacitinib 5 and 10 mg BID, respectively, vs 1.5 with placebo^32^; 2.7 and 4.3 with baricitinib 2‐ and 4 mg QD, respectively, vs 1.0 with placebo.^33^ HZ has also been reported in phase 3 RA studies of upadacitinib and filgotinib.^17^, ^18^ As such, the occurrence of HZ is likely a “class effect” of inhibiting at least JAK1,^34^ although viral reactivation could also be dependent on the overall impact of JAK inhibition.

Notably, IC_50_ values for filgotinib and its metabolite were not measurable for TPO, IL‐3, or GM‐CSF cytokine receptors (all JAK2‐dependent pathways). This may be related to the potency of filgotinib and the concentration range used in the assays. Overall, there was only minor differentiation between the effects of the JAK inhibitors on cytokine receptors mediated by JAK2/JAK2 pairs. However, tofacitinib and baricitinib appear to differentiate from each other clinically in terms of effect on platelet counts and hemoglobin levels, which could be related to the effects of JAK2 inhibition on TPO and EPO activity and production.^6^, ^35^, ^36^ In a pooled analysis of two LTE studies of tofacitinib 5 or 10 mg BID for RA, platelet counts initially decreased from baseline then stabilized over time, and hemoglobin levels increased from baseline over time.^37^ In the tofacitinib LTE study ORAL Sequel, increases in hemoglobin levels were observed from baseline to month 24, which then remained stable to month 96.^38^ In contrast, in an integrated analysis of phase 1b, phase 2, phase 3, and LTE RA studies, baricitinib 4 mg QD was associated with an initial increase in platelet counts, which then returned toward baseline; hemoglobin levels decreased from baseline to week 20, then returned to baseline or higher.^33^ Given that tofacitinib and baricitinib do not appear to differentially inhibit cytokine receptor signaling via JAK2 to a significant extent, alternative reasoning for the observed clinical difference may be important, for example, time course for inhibition. Indeed, time above IC_50_ may be significant.^39^

One observation with tofacitinib dosed QD vs BID suggests that some JAK effects may be more sensitive to daily drug holiday. In a phase 2 RA study, changes in levels of hemoglobin and neutrophils from baseline to week 24 were less pronounced with tofacitinib 20 mg QD (0.01 g/dL and −0.43 × 10^3^/mm^3^, respectively) vs 10 mg BID (−0.34 g/dL and −1.20 × 10^3^/mm^3^, respectively).^23^ While these differences are subtle, further clinical data are required to understand this phenomenon.

Lower levels of inhibition of JAK2‐dependent cytokine receptor signaling by tofacitinib, baricitinib, and upadacitinib have recently been reported,^40^ which contradict values reported here and in previous studies.^41^ However, whereas we used cytokine concentrations for all receptors that maximally induce STAT phosphorylation (>EC_95_), we note that McInnes et al^40^ utilized as much as 667‐fold lower concentrations of JAK2‐dependent cytokines. STAT phosphorylation can be more readily inhibited at lower cytokine concentrations (Figure S2), likely due to partial receptor activation, resulting in lower IC_50_. Physiologic cytokine concentrations vary, necessitating standardized cytokine receptor stimulation for proper comparison of inhibitor activity across different receptors. Similarly, the use of physiologic ATP concentration is important when comparing the inhibition potential of ATP‐competitive JAK inhibitors.^42^ The potency of upadacitinib against JAK enzymes measured in this study differs from those recently reported in Parmentier et al,^43^ which likely reflects differences in ATP concentrations used between studies.

Limitations of this analysis must be considered. The comparisons were based on doses of four JAK inhibitors which were considered to provide generally comparable clinical efficacy in patients with RA; these agents (and doses) have not all received regulatory approval. Furthermore, this analysis was limited to an extensive, but incomplete, list of cytokines; nevertheless, those evaluated represent key family members associated with each JAK pair, so the results are somewhat generalizable. While this analysis determined cytokine receptor inhibition profiles, the clinical impact of JAK selectivity could vary depending on factors such as patients' genetics or underlying inflammatory state (ie, comorbid diseases)^8^ as well as environmental factors or exogenous influences (eg, concomitant medications).

Research into inhibitors of alternative inflammatory pathways remains important. Indeed, second‐generation JAK inhibitors, preferentially selective for one JAK isoform, are being developed.^6^ For example, BMS‑986165 is a TYK2‐selective inhibitor under investigation for psoriasis^44^; and PF‐06651600 is a dual JAK3/TEC kinase‐selective inhibitor^45^ under investigation for alopecia (NCT02974868), Crohn's disease (NCT03395184), non‐segmental vitiligo (NCT03715829), RA (NCT02969044), and ulcerative colitis (NCT02958865). Possible differences in clinical profiles of compounds selective for TYK2 or JAK3 vs those selective for JAK1 are evidenced in early study data, although further research in more robust settings is required. For example, BMS‑986165 has not been associated with laboratory changes such as increased lipid levels,^44^ which can occur with IL‐6 inhibition.^46^

In conclusion, by applying an integrated modeling approach, the JAK inhibitors tofacitinib, baricitinib, upadacitinib, and filgotinib, at doses conveying reasonably equivalent clinical efficacy for RA, exhibited generally similar cytokine receptor inhibition profiles. Although some small numerical differences were observed, these do not appear to translate to significant differences in the JAK inhibitors' clinical profiles. At the same time, we appreciate that only robust clinical testing involving head‐to‐head studies may determine whether there are clinically meaningful differences between these JAK inhibitors. All JAK inhibitors, including novel second‐generation compounds with minimal JAK1 effects, need to be characterized via thorough preclinical, metabolic and pharmacological evaluation, adequate long‐term clinical data, and when available, real‐world experience.

## DISCLOSURES

All authors are employees and shareholders of Pfizer Inc. The authors report that this research did not receive external public or private foundation funding. The study was sponsored by Pfizer Inc.

## AUTHOR CONTRIBUTIONS

Participated in research design: Dowty, Lin, Jesson, Martin, Katkade, and Telliez.

Performed experiments: Dowty and Lin.

Performed data analysis: Dowty, Lin, Jesson, Hegen, Martin, Katkade, Menon, and Telliez.

Wrote or contributed to the writing of the manuscript: Dowty, Lin, Jesson, Hegen, Martin, Katkade, Menon, and Telliez.
