# Supplementary material for: Janus kinase inhibitors for the treatment of rheumatoid arthritis demonstrate similar profiles of in vitro cytokine receptor inhibition
Source: Pharmacol Res Perspect. 2019 Nov 15;7(6):e00537. doi: 10.1002/prp2.537 (PMC6857076; doi:10.1002/prp2.537)
Supplement: Supplementary file 1 [file PRP2-7-e00537-s001.pdf]

Figure S1: IC_{50} curves for representative cytokines from different receptor classes (results for one of the four experiments per cytokine)

A ***JAK1/JAK2 signaling pair***

IFNγ induced pSTAT1 - Donor 44 - Exp 1

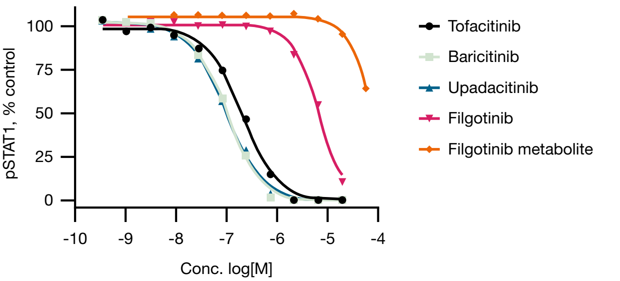

	Tofacitinib	Baricitinib	Upadacitinib	Filgotinib	Filgotinib metabolite
Bottom	= -0.2	= -0.2	= -0.2	= -0.2	= -0.2
Top	98.75	102.2	101.7	101	105.1
$LogIC_{50}$	-6.691	-7.012	-7.002	-5.17	-4.108
HillSlope	-1.248	-1.284	-1.171	-1.636	-1.77
IC_{50}	2.036e-007	9.724e-008	9.957e-008	6.757e-006	7.802e-005

B ***JAK1/TYK2 signaling pair***

IFNα induced pSTAT1 - Donor 256 - Exp 1

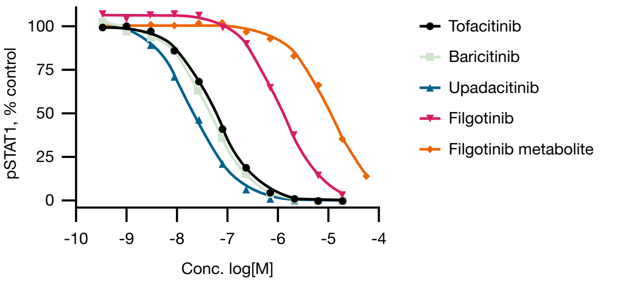

	Tofacitinib	Baricitinib	Upadacitinib	Filgotinib	Filgotinib metabolite
Bottom	= 0	= 0	= 0	= 0	= 0
Top	100.4	100.5	103.6	106.4	100.2
$LogIC_{50}$	-7.248	-7.339	-7.685	-5.931	-4.942
HillSlope	-1.055	-1.068	-0.9996	-1.07	-1.044
IC_{50}	5.653e-008	4.581e-008	2.068e-008	1.172e-006	1.143e-005

C ***JAK1/JAK2, JAK1/TYK2 signaling pair***

IL-6 induced pSTAT1 ($CD3^+$ cells) - Donor 44 - Exp 1

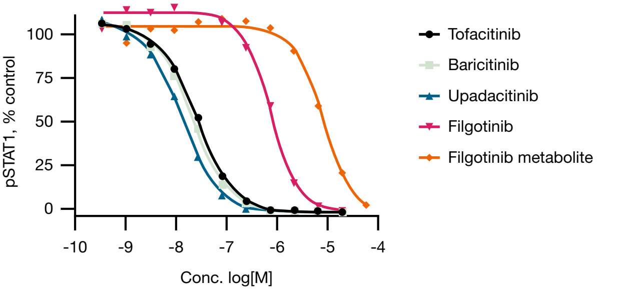

	Tofacitinib	Baricitinib	Upadacitinib	Filgotinib	Filgotinib metabolite
Bottom	= -2.5	= -2.5	= -2.5	= -2.5	= -2.5
Top	106.3	107.5	107.9	113.4	105.1
$LogIC_{50}$	-7.583	-7.687	-7.893	-6.12	-5.082
HillSlope	-1.152	-1.158	-1.157	-1.513	-1.513
IC_{50}	2.611e-008	2.057e-008	1.28e-008	7.589e-007	8.282e-006

D ***JAK1/JAK3 signaling pair***

IL-15 induced pSTAT5 - Donor 256 - Exp 1

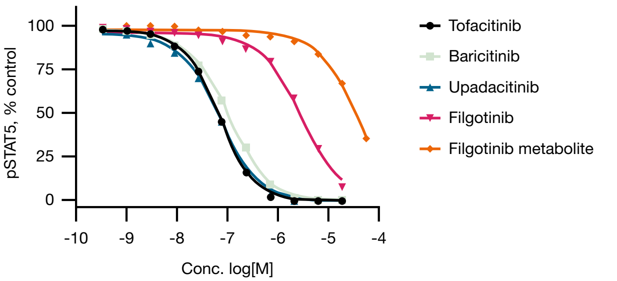

	Tofacitinib	Baricitinib	Upadacitinib	Filgotinib	Filgotinib metabolite
Bottom	= -0.1	= -0.1	= -0.1	= -0.1	= -0.1
Top	97.91	97.48	95.91	96.41	97.12
$LogIC_{50}$	-7.159	-6.957	-7.157	-5.526	-4.42
HillSlope	-1.261	-1.09	-1.122	-1.079	-1.086
IC_{50}	6.936e-008	1.105e-007	6.969e-008	2.979e-006	3.801e-005

E ***JAK1/TYK2 signaling pair***

IL-12 induced pSTAT4 - Donor 40 - Exp 1

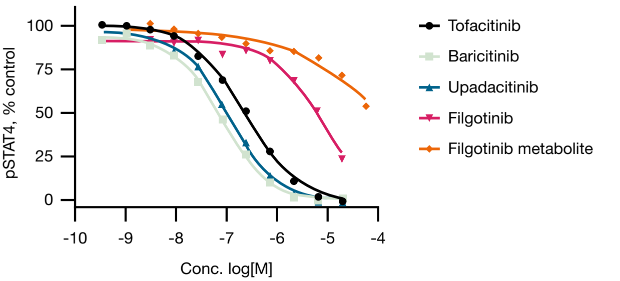

	Tofacitinib	Baricitinib	Upadacitinib	Filgotinib	Filgotinib metabolite
Bottom	= -0.6	= -0.6	= -0.6	= -0.6	= -0.6
Top	100.8	94.46	97.31	91.39	98.39
$LogIC_{50}$	-6.645	-7.096	-6.953	-5.125	-3.887
HillSlope	-0.8299	-0.918	-0.8971	-0.8843	-0.4573
IC_{50}	2.266e-007	8.024e-008	1.115e-007	7.506e-006	0.0001298

F ***JAK2/JAK2 signaling pair***

EPO induced pSTAT5 - Donor 573 - Exp 1

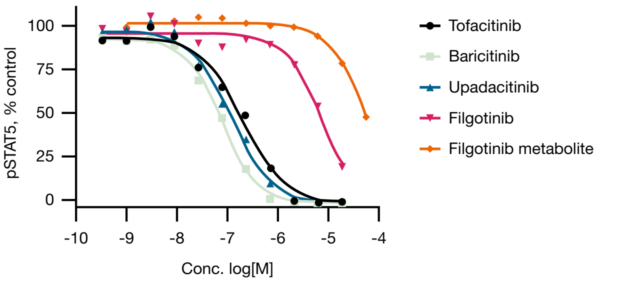

	Tofacitinib	Baricitinib	Upadacitinib	Filgotinib	Filgotinib metabolite
Bottom	= -0.9	= -0.9	= -0.9	= -0.9	= -0.9
Top	94.93	93.73	98.31	96.82	102.7
$LogIC_{50}$	-6.679	-7.102	-6.909	-5.121	-4.25
HillSlope	-1.038	-1.234	-1.065	-1.213	-1.2
IC_{50}	2.093e-007	7.908e-008	1.234e-007	7.577e-006	5.624e-005
