# Supplementary material for: Janus kinase inhibitors for the treatment of rheumatoid arthritis demonstrate similar profiles of in vitro cytokine receptor inhibition
Source: Pharmacol Res Perspect. 2019 Nov 15;7(6):e00537. doi: 10.1002/prp2.537 (PMC6857076; doi:10.1002/prp2.537)
Supplement: Supplementary file 2 [file PRP2-7-e00537-s002.pdf]

Figure S2: Mean IC_{50} values in human whole blood for tofacitinib using different IFNα concentrations

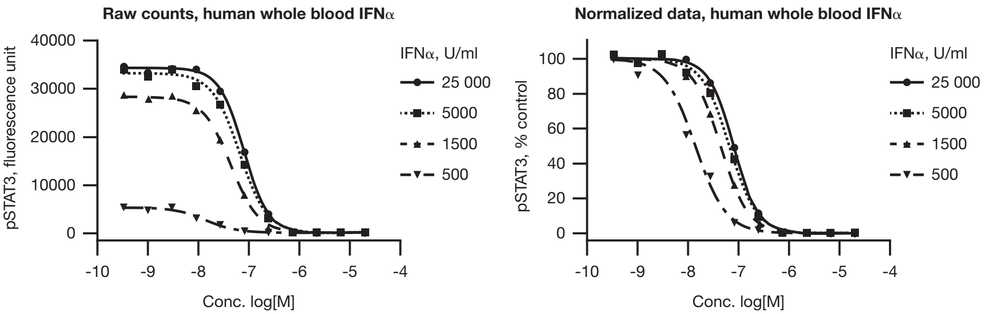

IFNα U/ml	EC_{xx}	Tofacitinib human whole blood IFNα IC_{50}, nM		
		Donor A	Donor B	Average
25 000	100	69	80	75
5000	95	62	66	64
1500	87	43	44	44
500	19	16	14	15
