# Supplementary material for: Janus kinase inhibitors for the treatment of rheumatoid arthritis demonstrate similar profiles of in vitro cytokine receptor inhibition
Source: Pharmacol Res Perspect. 2019 Nov 15;7(6):e00537. doi: 10.1002/prp2.537 (PMC6857076; doi:10.1002/prp2.537)
Supplement: Supplementary file 3 [file PRP2-7-e00537-s003.docx]

**FIGURE S1** IC_50_ curves for representative cytokines from different receptor classes (results for one of the four experiments per cytokine)


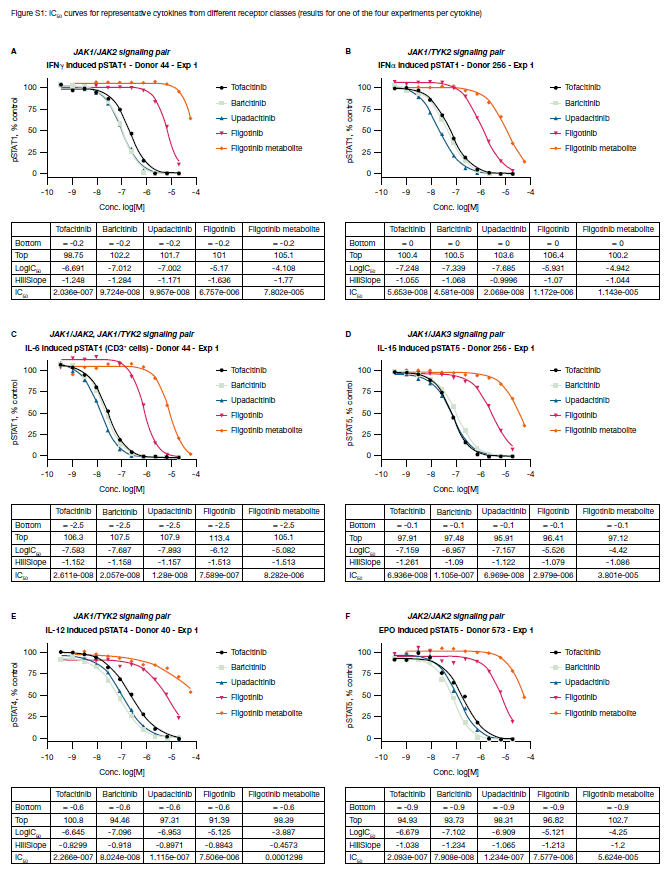


**FIGURE S2** Mean IC_50_ values in human whole blood for tofacitinib using different IFNα concentrations


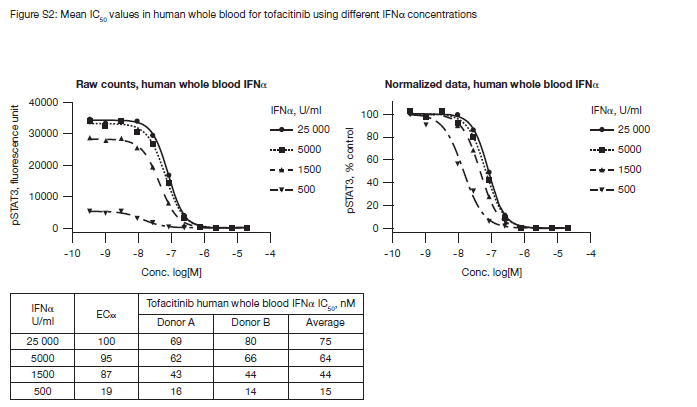


Shift of apparent IC_50_ using different concentrations of IFNα in a human whole blood assay. Human whole blood was collected from two healthy donors. A total of 90 µL/well of blood was added to 96-well polypropylene plates (Catalog No. 10755-246; VWR) and samples were pretreated with different concentrations of tofacitinib (0.0003–20 µmol/L) for 60 minutes at 37ºC. Then, IFNα (final 500, 1500, 5000, or 25 000 units/mL [U/mL]) was added to the blood samples and incubated for 15 minutes. The reaction was quenched by adding Phosflow Lyse/Fix Buffer 5X and incubated for 20 minutes at 37ºC. After removal of lysed erythrocytes, cells were permeabilized with ice-cold 90% methanol at 4ºC for 30 minutes. Finally, cells were incubated with anti-pSTAT3-AF647 antibody at 4°C overnight. Levels of pSTAT3 in the lymphocyte population were quantified by a flow cytometer. Fluorescence unit, which measures the level of pSTAT3, was calculated by multiplying the percent positive population and its mean fluorescence. Data from 11 tofacitinib concentrations (singlicate at each concentration) were normalized as a percentage of control, as shown in Equation 1 (see Methods section). Inhibition curves and apparent IC_50_ values were determined using Prism software. IFNα at 25 000 U/mL induced a maximal level of pSTAT3 (ie, 100 percent effective concentration [EC_100_]).
